# Long-lasting Insecticidal Nets to Prevent Visceral Leishmaniasis in the Indian Subcontinent; Methodological Lessons Learned from a Cluster Randomised Controlled Trial

**DOI:** 10.1371/journal.pntd.0003597

**Published:** 2015-04-09

**Authors:** Albert Picado, Bart Ostyn, Suman Rijal, Shyam Sundar, Shri Prakash Singh, François Chappuis, Murari Lal Das, Basudha Khanal, Kamlesh Gidwani, Epco Hasker, Jean Claude Dujardin, Veerle Vanlerberghe, Joris Menten, Marc Coosemans, Marleen Boelaert

**Affiliations:** 1 ISGlobal, Barcelona Center for International Health Research (CRESIB), Hospital Clínic—Universitat de Barcelona, Barcelona, Spain; 2 Department of Public Health, Institute of Tropical Medicine, Antwerp, Belgium; 3 Department of Medicine, B. P. Koirala Institute of Health Sciences, Dharan, Nepal; 4 Department of Medicine, Institute of Medical Sciences, Banaras Hindu University, Varanasi, India; 5 Department of Community Medicine, Institute of Medical Sciences, Banaras Hindu University, Varanasi, India; 6 Division of Tropical and Humanitarian Medicine, Geneva University Hospitals and University of Geneva, Geneva, Switzerland; 7 Department of Microbiology, B. P. Koirala Institute of Health Sciences, Dharan, Nepal; 8 Department of Biomedical Sciences, Institute of Tropical Medicine, Antwerp, Belgium; 9 Department of Biomedical Sciences, University of Antwerp, Antwerp, Belgium; Lancaster University, UNITED KINGDOM

In a recent paper, Nagpal et al. [1] voiced concerns about the limited or biased use of scientific evidence to support public health interventions to control neglected tropical diseases (NTDs). Visceral leishmaniasis (VL), also known as kala-azar, is one of the major NTDs and does not escape this problem. Transmission is vector-borne and the Indian subcontinent is the region reporting most of the VL cases worldwide. In this region, the main causative species is *Leishmania donovani* and *Phlebotomus argentipes* is the vector. Transmission is considered anthroponotic and peridomestic—occurring at night when female sand flies bite people sleeping inside their house. The World Health Organization and the governments of India, Nepal, and Bangladesh set out in 2005 to eliminate VL from the region by 2015 through a combination of early treatment of cases and vector control. However, while recent advances in diagnostic tools and drugs have significantly improved case management strategies, the available vector control tools against *P. argentipes* remain limited. The elimination initiative promotes the use of indoor residual spraying (IRS) of households and cattle sheds to reduce vector density, but the evidence underpinning the effectiveness of IRS in this region is scanty. Historical observations show that *L. donovani* transmission declined concomitantly with dichlorodiphenyltrichloroethane (DDT) spraying during the 1950s–60s to eradicate malaria. In the aftermath of this malaria eradication campaign, very few VL cases were observed in endemic regions until the mid-seventies, when there was resurgence of a VL epidemic in India [2]. To date, there are no randomized trials showing the effect of IRS on the incidence of clinical VL [3,4], though some studies showed a reduction in vector density. When the VL elimination initiative was launched in 2005, there were no clear alternatives for IRS as a vector control strategy. Insecticide treated nets (ITNs) were proposed as an alternative or complement to IRS on the basis of analogy arguments regarding their given efficacy against malaria [5] or on data from observational studies suggesting ITNs reduce the risk of VL [2]; but as for IRS, there were no randomized trials evaluating the effect of ITNs on *L. donovani* transmission. In this context, a number of field studies were conducted in the Indian subcontinent in the past decade to evaluate the effectiveness and impact of ITNs and other vector control tools on VL. Most of these studies have been reviewed in detail in two recent papers [3,4]. The only two studies evaluating the impact of vector control interventions on clinical outcomes found conflicting results. First, the KALANET project, a cluster randomised controlled trial (CRT) in India and Nepal, showed that mass-distribution of ITNs did not reduce the risk of *L. donovani* infection or clinical VL [6]. Then, an intervention trial in Bangladesh suggested that widespread bed net impregnation with slow-release insecticide may reduce the frequency of VL [7]. Technical (e.g., type of nets and insecticides, lack of replicas and randomisation in Bangladesh) and biological factors (e.g., insecticide susceptibility and sand fly behaviour) may explain the different results observed. This apparent contradiction raises the question about the role that ITN may play in controlling VL in the Indian subcontinent but has also triggered a lot of discussion on methodology and evidence levels required when evaluating vector control tools for VL. In this paper, we would like to summarise the lessons learned from the KALANET CRT in terms of methodology to inform the generation of future evidence and discuss interpretation of findings against this background.

The KALANET trial was designed to evaluate the distribution of ITNs as a public health intervention to prevent VL in the Indian subcontinent. The objective was to answer the question: “in the current context in India and Nepal, would free mass distribution of ITN significantly reduce the incidence rate of VL in endemic regions?” This was a question asked not at the individual level, whether sleeping under an ITNs protects an individual against VL compared to an individual not sleeping under an ITN, but a question asked at program level: does such prevention measure reduce VL incidence in communities. Furthermore, the question was about effectiveness in real life conditions and not a question about efficacy of ITNs in “laboratory conditions,” a difference that is clear for public health experts but not necessarily for all readers. The answer to this question may, for instance, be entirely different in a country such as Bangladesh, where no vector control program was operating for many years, no spraying was implemented at the time, and fewer households were using untreated nets, compared to India and Nepal. The first lesson we learned was about the importance of clarifying the research question itself.

To answer the above question, we adopted a study design used previously in a successful intervention trial on zoonotic VL transmission [8]. We designed a CRT to demonstrate a 50% reduction on the risk of *L. donovani* infection associated to the village-wide distribution of ITN [6]. Long-lasting insecticidal nets (LNs) were chosen as intervention as they remain effective for three to four years in the field [2]. Incident *L. donovani* infection, measured as seroconversion in the Direct Agglutination Test (DAT), was used as the main outcome. Measuring the impact of LN on the risk of clinical VL would have been the preferred primary outcome, but its low incidence and the long incubation period precluded this. Incidence of VL cases was nonetheless measured as secondary outcome. The trial was conducted in 26 high-incidence clusters (16 in India and 10 in Nepal) with over 20,000 inhabitants followed over 24 months. After randomisation, LNs were distributed in all households in the 13 intervention clusters, with the number of LNs proportional to household size, to make sure that all household members could sleep under the nets. Participants in the control clusters were allowed to continue using their untreated nets. The effect of LNs on the incidence rate of seroconversion and VL was compared after 24 months between intervention and control clusters. No LNs were used in the control clusters. The results of the trial, analysed as suggested by Hayes and Moulton [9], showed that the large scale distribution of LN did not reduce the risk of *L. donovani* infection [6]. These results were consistent across several endpoints measured, as no difference was observed in (1) incidence rate of clinical VL [6], (2) seroconversion in rK39 ELISA (a second serological marker) [10], and (3) mean *P. argentipes* exposure measured at cluster level by a sand fly saliva antibody detection ELISA [11]. Similarly, the reduction of *P. argentipes* density indoor in the study clusters was limited (24.9%) [12]. The main conclusion of the trial was that “there is no evidence that using LNs as a public health intervention provides additional protection against VL at community level compared with existing control practices in India and Nepal (e.g., irregular use of untreated nets and IRS). This does not mean that the use of LNs in those VL endemic regions should be dismissed, as they may provide some degree of personal protection against sand flies [13] and have been shown to reduce the risk of malaria [6]. However, the VL elimination initiative in India and Nepal cannot rely on the stand-alone use of LNs to effectively control transmission.”

The above message is complex and was disappointing for many, in the first place for the researchers themselves, as the hopes for a user-friendly, household-controlled tool to control VL in the Indian subcontinent were given a serious blow. Moreover, results from a negative trial are hard to communicate. Criticism of peers focused on four main areas: 1. the biological rationale for the intervention, 2. the choice and number of units of analysis, 3. the choice of endpoint and 4. the adherence to the intervention. Stockdale and Newton also identified these methodological issues as key factors to evaluate studies testing preventative methods against human leishmaniasis infection [3].

## Rationale for the Intervention

In theory, LNs could be an effective tool to prevent *L. donovani* transmission, as *P. argentipes* are supposed to bite people indoors while they sleep [14]. However, recent entomological findings in India indicate that *L. donovani* vectors are more exophilic and exophagic than previously reported [15,16]. If *P. argentipes* bite people outdoors (e.g., in the early evening when and where bed nets are not deployed), LNs will have a limited impact on *L. donovani* transmission. Moreover, as *P. argentipes* is also zoophagic [17], LNs will have a limited impact on vector survival and thus on transmission [18]. We hypothesised that these were the main factors explaining the KALANET trial results as participants used the mosquito nets correctly (see below); vectors were susceptible to the insecticide used in the nets (e.g., deltamethrin [19]), and LNs provide an effective barrier effect against *P. argentipes* [20]. Unfortunately, the KALANET trial was not designed to study *P. argentipes* behaviour. For example, the effect of LNs on vector density was only measured indoors [21]. Entomological studies are urgently required to document the transmission dynamics of *L. donovani* in the Indian subcontinent.

## The Unit of Analysis

In KALANET, the unit of analysis was a cluster with 350 to 1,500 people corresponding to a hamlet (“tola” in India, “ward” in Nepal). These clusters were selected based on their previous history of VL: at least one VL case in the last three years and minimum VL incidence of 0.8% during that period. Clusters were pair-matched based on their prior VL incidence. The number of clusters was calculated assuming a 2% yearly *L. donovani* infection incidence rate and a coefficient of variation between clusters (κ) of 0.25 [6]. It is known that VL cases are clustered in space and time. VL cases tend to occur in microepidemics, affecting one village, lasting three to five years, fading out only to reappear in another area. This phenomenon is supposed to be related, among other factors, to herd immunity at village level. The criteria used to select the clusters in the KALANET study may have resulted in a variety of villages at different stages in those “microepidemic cycles,” with some villages still on the increasing slope of the incidence curve and others on the decrease. This did not invalidate the study design or trial outcomes, as clusters were randomly allocated to both study arms. However, the inclusion of some clusters in the late phases of the local epidemic may have decreased the power of the study as more incident infections and VL cases are expected in a more “naïve” population. Future trial designs should take this into account and try to include clusters as early in the cycle as possible. Sample size calculations at design stage were based on an expected *L. donovani* infection incidence of 2% which proved correct—but we underestimated the clustering of incident infections as the observed coefficient of variation for *L. donovani* infection was 0.56 instead of 0.25. This observed k-value may be of use for the planning of future intervention trials. In the KALANET trial, we increased the number of clusters by 30% (13 clusters per arm instead of 10 initially planned) to increase the power of the study.

## The Endpoint

Using VL cases as the primary outcome for a community intervention trial, instead of *L. donovani* infection, is the better option. However, this would have necessitated a much larger number of clusters, and the low VL incidence may result in clusters having low counts (e.g., less than five VL cases). We therefore considered several *L. donovani* infection markers as alternatives. Seroconversion in the DAT test is strongly associated with clinical VL [22,23]. We chose it as the main outcome of the KALANET trial based on results from previous VL trials [8]. rK39 ELISA, used as a secondary serological marker, showed poor agreement with DAT [24] and presented different kinetics in past VL cases compared to DAT [25]. High rK39 titres are equally associated with progression to clinical disease [22]. The Leishmanin Skin Test (LST), initially postulated as an alternative or complement to serological tests, was discarded because of problems from a source good manufacturing practices (GMP)-manufactured antigen and some erratic results observed in the study area [26]. As stated above, analysis of all endpoints in KALANET gave consistent results.

## Adherence to the Intervention

From the start of the study project, we included a large research component on “acceptability” of the intervention, using mixed methods, including Knowledge-Attitude-Practices surveys, observation, and focus group discussions. The acceptability of bed nets was a priori not considered problematic in this region as bed net coverage and use is high in rural villages in the Indian subcontinent [7,27,28]. People like to protect themselves from insect nuisance at night by sleeping under nets, as it enhances quality of sleep. This pattern is season dependent though, with less use in the hotter months. The availability of commercial bed nets in the communities living in the KALANET clusters was widespread before we started the trial: 70% to 80% of households in Nepal and India had at least one net at baseline [6]. Those nets were all untreated, many were damaged, and most of the families did not have enough nets to protect all household members. Nevertheless, untreated mosquito nets were commonplace in the study villages and most households used untreated nets [27,28].

However, even if communities in the study area were familiar with the use of bed nets, we conducted a series of activities to ensure the correct and regular use of LNs distributed in intervention clusters. First, we selected from the available LN brands the product that best met the people’s preference based on a formal comparative evaluation of several brands [29], and we took into account cultural preferences regarding colour and size of the nets. Enough LNs were provided per household to ensure all family members could sleep under a treated net while at the same time respecting existing sleeping patterns. We did not take away the existing commercial nets in the control clusters, but did so in the intervention clusters, in exchange for the new LNs. It is important to remember that untreated nets were already in use before the trial with no apparent effect on *L. donovani* transmission in those VL endemic communities. Forbidding the use of untreated nets in control clusters would have been unethical.

To enhance the correct use of LNs, field workers organised meetings in the villages and distributed Information-Education-Communication (IEC) materials (e.g., pictorial diagrams in local language) to promote the correct use (e.g., net deployment, washing frequency) of the LNs. The content of these IEC messages was largely inspired from prior findings in focus group discussions on perception of the disease and attitudes with regard to preventive measures. Finally, quarterly house-to-house surveys were conducted during the trial to monitor and promote the regular use of LN in the intervention clusters. So by the end of the trial, the use of LNs in intervention clusters was very high: 91% of the individuals in those clusters slept more than 80% of the nights under a treated net [6]. This figure contrasts with the 30% of people in control clusters, where no LNs were distributed, who reported regular use of their untreated nets during the trial [6]. Some peer reviewers argued that the use of untreated nets in control clusters may have masked the possible effect of LNs in VL incidence, and this is correct to a certain extent, but cannot have led to a huge impact given their low, irregular, and inconsistent use.

The KALANET study was a huge collaborative endeavour of seven research teams in six countries, conducted at a marginal cost of 2 million €, a budget provided by the 6^th^ Framework Programme of the European Union (INCO/RTD). One can ask whether this huge research effort is (a) required to underpin public health policy and (b) cost-effective?

We think that this level of evidence from properly conducted randomized controlled trials testing effects on human morbidity and mortality is indeed required before adopting novel vector control tools as public health policy, as effects on vector density only are not sufficient to demonstrate health impact. A randomized controlled design over a sufficient number of study units is required in this highly variable and hyper-clustered disease. CRTs remain the preferred design to evaluate public health interventions at community level.

Whether these large research projects are also “cost-effective” or value for money is not easy to answer, as the opportunity cost of ineffective health policy should be put in the balance. Moreover, the benefits of a CRT are often not limited to a single outcome measure. The KALANET study allowed us to better understand the epidemiology of VL in India and Nepal, and the data generated were used to develop a mathematical model evaluating the *L. donovani* transmission parameters and control measures against VL in the Indian subcontinent [30,31]. This transmission model suggests that integrated vector management (e.g., combining IRS and LNs) is the best approach to overcome the limitations of the current vector control strategy [31]. Mathematical modelling can help designing new vector control methods that then need to be evaluated in the field. The KALANET project not only highlighted the huge need for innovation in vector control in VL, but pointed also to the fundamental knowledge gaps in this domain. Better understanding of the vector bionomics (e.g., biting rhythm, population dynamics, endophagy and exophagy, and endophily and exophily) and human behaviour (e.g., sleeping habits) is essential to develop new control measures.

Nonetheless, if we want to foster innovation in vector control, we should scrutinize and simplify the evaluation methodology of new vector control tools for VL, as the huge resources and time required for the comprehensive evaluation approach adopted in the KALANET project cannot be mainstreamed for every single study. As for drugs and diagnostics, a “pipeline approach” to the development of new vector control interventions, with proof of principle leading to evaluation in several staged design phases (I, II, III, and IV), should be promoted [32]. Alternative methods for a quick evaluation of the entomological efficacy of new *P. argentipes* control tools under field conditions are needed [13]. An individual marker of *P. argentipes* exposure as a sand fly saliva antibody test—if validated—could allow the direct evaluation of vector control measures. For the last stage of the pipeline, the impact evaluation, alternative CRT designs (e.g., crossover or stepped wedge designs) and sample size calculation methods should be explored to take into account the spatiotemporal clustering of rare events. We also suggest selecting clusters in the early stages of the local epidemic cycle and using a higher k-value (e.g., 0.50) for sample size calculation as well as analysis. The use of new end points to measure the impact of those interventions at population level in a more efficient way would make a difference [33]. New and better markers of *L. donovani* infection (e.g., cellular immunity markers) should be developed and evaluated.

In conclusion, the KALANET trial is thus far the only CRT evaluating the impact of ITN on VL. The CRT design should be taken into account when the KALANET results are evaluated and compared to other studies using a less robust methodology (e.g., a nonrandomized trial comparing two clusters, one area with bed net impregnation and one without in Bangladesh [7]). In the context of VL in the Indian subcontinent, entomological and epidemiological studies should be conducted to better understand *L. donovani* transmission in endemic villages. IRS, which remains the main vector control strategy in the region, needs to be reassessed, and integrated vector methods (e.g., IRS combined with LNs) should be evaluated using a CRT design. Finally, a randomized controlled design is essential to produce evidence for health policy in this field, and methodological innovation is urgently needed to make the Research & Development (R&D) pipeline process more efficient.
